# The association between oxidative balance scores and all-cause mortality and cancer-specific mortality in cancer survivors: a retrospective cohort study

**DOI:** 10.3389/fnut.2025.1522048

**Published:** 2025-05-21

**Authors:** Ran He, Qilei Zhu, Youjun Ye, Qingyu Tu, Jiayao Yang, Shuaihang Chen, Qiannan Liu, Changsheng Xie

**Affiliations:** ^1^The First School of Clinical Medicine, Zhejiang Chinese Medical University, Hangzhou, China; ^2^The Third Clinical Medical College, Zhejiang Chinese Medical University, Hangzhou, China; ^3^Ningbo TCM Hospital of Zhejiang Chinese Medical University, Ningbo, China; ^4^Jiangnan Hospital Affiliated to Zhejiang Chinese Medical University, Hangzhou, Zhejiang, China; ^5^The Second Clinical Medical College of Zhejiang Chinese Medical University, Hangzhou, China; ^6^Department of Medical Oncology, The First Affiliated Hospital of Zhejiang Chinese Medical University, Zhejiang Provincial Hospital of Traditional Chinese Medicine, Hangzhou, China

**Keywords:** OBS, cancer survivors, all-cause mortality, cancer-specific mortality, retrospective cohort study, NHANES

## Abstract

**Background:**

Numerous studies have established that oxidative stress significantly affects the long-term survival of cancer survivors. However, there is currently no comprehensive measure to assess oxidative stress levels in these individuals that associates with all-cause, cause-specific, and cardiovascular disease (CVD) mortality. This study aims to investigate the relationship between Oxidative Balance Score (OBS) in American cancer survivors and their risks of all-cause, cancer-specific, and CVD mortality.

**Methods:**

This research included cancer survivors from the National Health and Nutrition Examination Survey dataset covering the 2001–2018 cycles, incorporating appropriate weighting. The OBS, a composite index reflecting oxidative stress status, was constructed based on 16 dietary components and 4 lifestyle factors, with higher OBS indicating greater antioxidant capacity. Using multivariable Cox regression, restricted cubic splines analysis (RCS), subgroup analysis, and sensitivity analysis, we examined the associations between OBS and all-cause, cancer-specific, and CVD mortality, including further stratified analyses for specific cancer types and populations.

**Results:**

The study enrolled 2,131 eligible cancer survivors, with a median follow-up of 115 months and 673 recorded deaths. Weighted multivariable Cox regression results showed that each unit increase in OBS was associated with a 3% decrease in all-cause mortality (Hazard Ratios [HR]: 0.97, 95% Confidence interval [CI]: 0.95, 0.99, *p* = 0.006). Among participants, those in the highest OBS quartile (Q4) had a 40% lower risk of all-cause mortality compared to those in the lowest quartile (Q1) (HR: 0.60, 95% CI: 0.41, 0.88, *p* = 0.009). A similar significant association was found with cancer-specific mortality, while no significant association was noted for CVD mortality. RCS analysis further highlighted a significant linear negative association. Subgroup analyses indicated stronger associations with all-cause and cancer-specific mortality among breast cancer patients, those without stroke or arthritis individuals. Sensitivity analysis confirmed the robustness of these findings.

**Conclusion:**

The study reveals a significant linear negative association between OBS in cancer survivors and both all-cause and cancer-specific mortality.

## Introduction

Improving the survival outcomes of cancer survivors and preventing adverse events is a significant challenge for global public health. Research indicates that the incidence of new cancer cases worldwide is on the rise, with projections suggesting that there will be 2 million newly diagnosed cancer cases and 600,000 cancer deaths in the United States by 2025 (1). However, advancements in medical care have led to a gradual decrease in cancer mortality rates (2). This trend has resulted in a growing population of cancer survivors, with estimates suggesting that by 2040, the number of cancer survivors globally could exceed 26 million (3). While developments in cancer diagnosis and treatment have improved short-term survival for patients, long-term survival remains fraught with numerous risk factors for cancer survivors (4). On one hand, residual cancer cells and the disruption of immune microenvironment balance can lead to persistent, systemic, and debilitating complications, such as chronic inflammation, electrolyte imbalances, and cachexia (5). On the other hand, the side effects of long-term cancer treatments, such as chemotherapy and radiation, significantly impact the quality of life for cancer survivors (6). Studies show that these risks affecting long-term survival are closely linked to individual dietary patterns and lifestyle habits (7). Given the many challenges faced by cancer survivors in achieving long-term survival, it is clinically significant to explore the potential factors influencing their outcomes through a comprehensive understanding of dietary habits and lifestyle choices.

Recent studies have shown that redox balance plays a crucial role in the long-term survival of cancer survivors (8). When the balance between oxidants and antioxidants in the body is disrupted, oxidative stress occurs, causing an excessive buildup of reactive oxygen species (ROS). These ROS can abnormally trigger various transcription factors, interfere with the proper functioning of tumor suppressor genes, or weaken the tumor immune microenvironment—ultimately enabling cancer cells to evade immune surveillance and driving tumor growth and spread (9–11). Moreover, previous research indicates that high levels of oxidative stress can also hasten aging and mortality while contributing to the onset of various diseases, including diabetes, cancer, and cardiovascular conditions (12, 13). Thus, for cancer survivors, a disruption in oxidative balance not only increases the risk of cancer mortality but is also associated with heightened risks of heart disease, diabetes, stroke, and other fatal threats, significantly impacting long-term survival.

Factors such as physical activity, diet, smoking, and other lifestyle habits can all influence the body’s level of oxidative stress. Since no single factor can fully capture the complexity of redox balance, researchers developed the Oxidative Balance Score (OBS)—a composite measure that integrates 16 dietary and 4 lifestyle components into a unified score. A higher OBS suggests a more favorable oxidative balance (14). Prior research has linked higher OBS values to reduced risk of several conditions, including depression, cardio-renal-metabolic syndrome, and abdominal aortic calcification (15–17).

The relationship between redox balance and long-term survival risk for cancer survivors remains to be fully elucidated, and there is a lack of comprehensive clinical studies that analyze and discuss this issue in depth. Therefore, exploring the long-term survival risks of cancer survivors through the lens of OBS may provide innovative insights for clinical diagnosis, treatment, and disease prevention. This study incorporates relevant data from cancer survivors in the National Health and Nutrition Examination Survey (NHANES) database from 2001 to 2018 and aims to evaluate the association between OBS and all-cause mortality, cancer-specific mortality, and cardiovascular disease (CVD) mortality in a large retrospective cohort study.

## Method

### Data source and research population

This study included data from 9 survey cycles of the NHANES spanning 2001–2018. Each cycle consisted of a continuous 2-year rolling survey, recruiting nationally representative samples to ensure the temporal continuity of the data and the representativeness of the population. These 9 cycles were: 2001–2002, 2003–2004, 2005–2006, 2007–2008, 2009–2010, 2011–2012, 2013–2014, 2015–2016, and 2017–2018. The data used in this study are all publicly available from the official NHANES website.^1^ The NHANES employs a stratified, multistage, and complex probability sampling design. All survey protocols were approved by the Ethics Review Board of the National Center for Health Statistics (NCHS), and all participants provided written informed consent. The following are the protocol approval numbers provided by NCHS: Protocol #98-12 (NHANES 1999–2004), Protocol #2005-06 (NHANES 2005–2006), Continuation of Protocol #2005-06 (NHANES 2007–2010), Protocol #2011-17 (NHANES 2011–2012), Continuation of Protocol #2011-17 (NHANES 2013–2016), Continuation of Protocol #2011-17 & Protocol #2018-01 (NHANES 2017–2018). This study utilized publicly available NHANES data to conduct a retrospective cohort study. The study design adhered to the relevant specifications of the Strengthening the Reporting of Observational Studies in Epidemiology (STROBE) guidelines.

**Figure 1:**
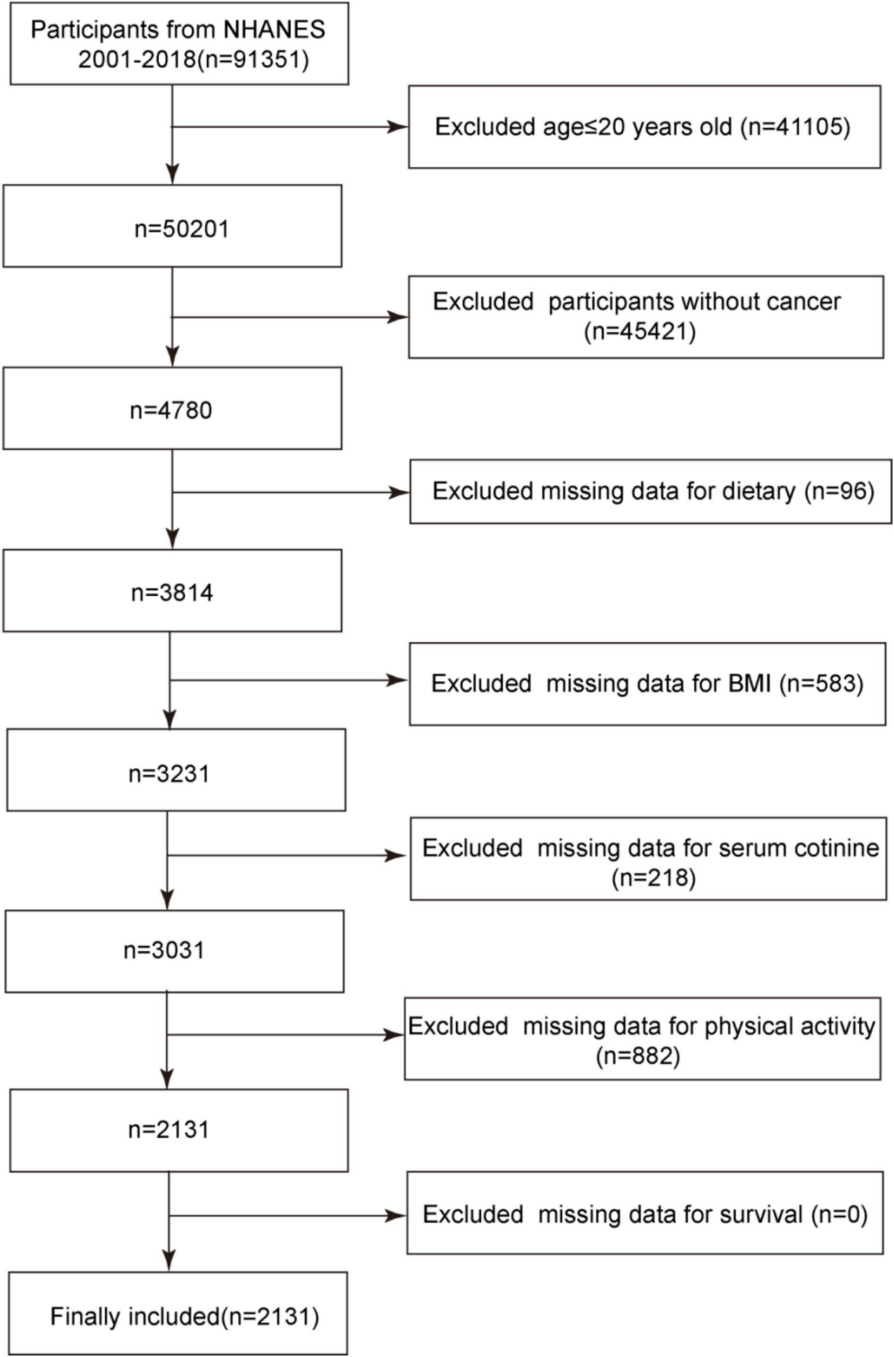
Study flowchart.

The study population consisted of cancer survivors with complete data from the NHANES database. In total, there were 91,351 participants in the 2001–2018 cycles of the NHANES database. Initially, 41,105 participants under the age of 20 were excluded. NHANES defines adults as individuals aged 20 years and above, and key variables used in constructing the OBS—such as smoking history and alcohol consumption—can be reliably and consistently obtained only in this age group. Including children and adolescents (under 20 years), who differ in both physiology and disease etiology, would introduce variability in the measurement of exposures and outcomes. Furthermore, since this study focuses on cancer survivors, the vast majority of whom are adults, limiting the analysis to participants aged 20 years or older helps maintain the relevance and consistency of the OBS assessment for the target population. Subsequently, based on results from the MCQ220 questionnaire within NHANES, 45,421 non-cancer patients were further excluded, leaving 4,780 participants. After excluding those missing dietary data, body mass index (BMI), serum cotinine, weekly physical activity, and daily alcohol consumption—key components of the OBS—2,131 participants remained. All of these participants had complete follow-up data, resulting in a final study population of 2,131 cancer survivors. The specific screening process is illustrated in Figure 1.

### Definition of OBS

The OBS is a composite measure that integrates 16 nutrient-based and 4 lifestyle-related variables to reflect an individual’s oxidative stress status (14, 18). Higher OBS values correspond to greater antioxidant potential, it may serve as a marker of redox balance, potentially useful in identifying high-risk individuals and evaluating the effectiveness of behavioral or nutritional interventions in clinical and public health settings. The score comprises 15 antioxidant and 5 pro-oxidant components. All variables were sex-stratified and categorized using tertiles or standardized thresholds. Antioxidant factors were assigned scores of 0, 1, or 2, whereas pro-oxidant components were scored inversely. Lifestyle-related components included alcohol consumption, smoking status (assessed by serum cotinine), physical activity, and BMI. Alcohol intake was categorized as follows: non-drinkers received 2 points, light drinkers (men: 0–30 g/day, women: 0–15 g/day) received 1 point, and heavy drinkers (>30 g/day for men, >15 g/day for women) received 0 points. Physical activity was assessed using weekly MET-minutes based on NHANES-derived exercise intensity and frequency. Smoking exposure was evaluated by serum cotinine levels, which captured both active and passive smoking. BMI was scored according to sex-specific tertiles. A detailed description of the scoring methodology is provided in Supplementary Table 1.

### Definition of outcomes

The primary outcome measures in this study were all-cause mortality, cancer-specific mortality, and CVD mortality among participants. The National Death Index (NDI) database prospectively recorded follow-up information for certain participants from the NHANES database up to December 31, 2019, including details on survival status, causes of death, and survival duration. Causes of death were classified using the International Classification of Diseases, 10th Revision (ICD-10), where ICD-10 codes I00-I09, I11, I13, I20-I51, and I60-I69 were categorized as CVD mortality, while ICD-10 codes C00-C97 were classified under cancer-specific mortality.

### Covariates

To minimize the impact of potential confounding factors on the study results, this research comprehensively included various covariates. From a demographic perspective, the study accounted for age, sex, race, marital status, education level, household income, and poverty index. In terms of health status, the presence of conditions such as alcohol consumption, smoking history, hypertension, diabetes, stroke, coronary heart disease, heart failure, and arthritis was considered. Additionally, participants’ estimated glomerular filtration rate (eGFR) was calculated using the CKD-EPI 2009 equation to reflect kidney function (19). The study also accounted for potential confounding arising from differences in daily energy intake. Specifically, a alcohol consumption was defined as consuming at least 12 alcoholic drinks in the past year, a smoking history was defined as having smoked more than 100 cigarettes in the past, and a poverty index ratio (PIR) of less than 1.3 was classified as low income, between 1.3 and 3.5 as middle class, and above 3.5 as high income. Previous research has indicated that a waist-to-height ratio (WtHR) above 0.5 is indicative of abdominal obesity, which is more advantageous than BMI in predicting cardiovascular risks associated with obesity (20). Therefore, this study included this metric to assess participants’ abdominal obesity.

### Statistical analyses

To minimize the influence of potential confounding factors on the study results, this research comprehensively included covariates. Participants were weighted according to NHANES database recommendations, reflecting the U.S. population accurately due to the multi-stage complex sampling method used in NHANES. This study utilized dietary data from the DR1TOT item in the dietary questionnaire, which captures total nutrient intake. The analysis was weighted using WTDRD1, following the weighting method recommended by the NHANES database. To address the minimal missingness observed in some covariates and avoid loss of statistical power or potential bias from listwise deletion, we applied multiple imputation using the mice package in R. A chained equations model was implemented under the Predictive Mean Matching (PMM) framework, allowing each variable with missing data to be imputed based on its specific distribution and relationship with other covariates. Five imputed datasets were generated and combined using Rubin’s rules to reduce variability introduced by single imputation. The imputation model included key covariates such as the poverty index, eGFR, and medical history variables including hypertension, stroke, arthritis, heart failure, coronary heart disease, smoking, and alcohol consumption. The highest missingness was observed in alcohol consumption (13.13%) and PIR (7.03%), while missingness for all other variables remained below 2%. Continuous variables were expressed as means (standard deviations) if they followed a normal distribution, assessed with a weighted Student’s t-test, and as medians (interquartile ranges) if not, using a weighted Kruskal-Wallis test. Categorical variables were presented as numbers (percentages) and analyzed using weighted chi-squared tests. Following statistical methods from prior literature (21), OBS was divided into four quartiles: Q1 (1–14), Q2 (15–20), Q3 (21–26), and Q4 (27–37) for further comparison.

To accurately assess the association between OBS and all-cause mortality, cancer-specific mortality, and CVD mortality in cancer survivors, a weighted multivariable cox proportional hazards model was employed to calculate hazard ratios (HR) and 95% confidence intervals (CI). Three models were used: the Unadjusted Model with no covariate adjustments, Adjusted Model 1 which adjusted for general demographic characteristics (age, sex, race, marital status, education, and PIR), and Adjusted Model 2, the fully adjusted model, which added adjustments for eGFR, energy intake, alcohol consumption, smoking history, WtHR, and comorbidities such as hypertension, diabetes, stroke, coronary heart disease, heart failure, and arthritis. The covariates included in the adjusted models were chosen based on previous studies and clinical relevance (21–23). Additionally, subgroup analyses were conducted for cancer types with larger sample sizes.

Survival differences among cancer survivors across different OBS quartiles were analyzed using weighted Kaplan–Meier (KM) curves. Weighted restricted cubic splines (RCS) were employed to assess the linear or nonlinear associations between OBS and all-cause mortality, cancer-specific mortality, and CVD mortality in Adjusted Model 2. The optimal number of knots for the RCS analysis was determined using the Akaike information criterion to achieve the best-fitting model. To evaluate the robustness of the observed association across diverse populations, we performed weighted stratified analyses based on key subgroup characteristics, including sex, marital status, PIR, abdominal obesity, and a history of hypertension, diabetes, stroke, coronary heart disease, heart failure, or arthritis. We assessed potential effect modification by estimating the association separately within each subgroup, aiming to determine whether the strength of the association differed according to individual-level factors. Finally, to assess the stability of our findings, we performed several sensitivity analyses: 1. We excluded cancer survivors who died within the first two years of follow-up to reduce potential reverse causality, ensuring that the OBS reflected a pre-existing physiological state rather than a terminal-phase artifact. 2. Individuals with extreme OBS values (top and bottom 2.5%) were excluded to mitigate the influence of outliers and enhance analytical robustness. 3. Participants with non-solid malignancies (e.g., leukemia, lymphoma) were removed to ensure they would not be the source of clinical heterogeneity. 4. We re-evaluated Adjusted Model 2 using the Fine-Gray competing risk model to account for competing risks between cancer-related and cardiovascular mortality. This model allows for more accurate estimation of cause-specific mortality risks by incorporating the presence of multiple competing death causes, avoiding bias introduced by simply excluding other outcomes.

Statistical analyses were performed using R version 4.2.3, with *p* < 0.05 considered statistically significant.

## Results

### Study population

This study included a total of 2,131 cancer survivors from the NHANES database covering the period from 2001 to 2018, with a weighted population of 11,616,181. The median age of the participants was 67 years, with males comprising 50.07% of the population. The median follow-up time for cancer survivors was 115 months, during which 673 deaths were observed, including 205 cancer-specific deaths and 146 CVD deaths. Cancer survivors in the highest OBS quartile (Q4) were more likely than those in the lowest quartile (Q1) to hold a college degree or higher, to be married or cohabiting, to have a higher PIR, to be without abdominal obesity, to have higher daily energy intake, and to have higher eGFR levels. Additionally, they were less likely to have smoking history, coronary heart disease, stroke, or hypertension, and they had a lower probability of experiencing death or cancer-specific death events. All these differences were statistically significant (*p* < 0.05). However, there was no significant difference in the probability of cardiovascular death (*p* > 0.05). Detailed baseline characteristics are presented in Table 1.

**Table 1:** Baseline study population characteristics.

Characteristic	Total (*n* = 2,131)	Q1 (4–14, *n* = 537)	Q2 (15–20, *n* = 491)	Q3 (21–26, *n* = 556)	Q4 (27–37, *n* = 547)	*p*-value
Sex (%)						0.226
Male	1,067 (50.07%)	269 (50.09%)	240 (48.88%)	292 (52.52%)	266 (48.63%)	
Female	1,064 (49.93%)	268 (49.91%)	251 (51.12%)	264 (47.48%)	281 (51.37%)	
Age (year)	67.00 [55.00, 76.00]	66.00 [54.00, 75.00]	68.50 [55.75, 77.00]	68.00 [57.00, 77.00]	67.00 [55.00, 76.00]	0.062
Race (%)						0.006
Mexican American	126 (5.91%)	33 (6.15%)	33 (6.72%)	30 (5.40%)	30 (5.48%)	
Other Hispanic	92 (4.32%)	19 (3.54%)	25 (5.09%)	27 (4.86%)	21 (3.84%)	
Non-Hispanic White	1,617 (75.88%)	370 (68.90%)	369 (75.15%)	440 (79.14%)	438 (80.07%)	
Non-Hispanic Black	226 (10.61%)	96 (17.88%)	46 (9.37%)	45 (8.09%)	39 (7.13%)	
Other Race	70 (3.28%)	19 (3.54%)	18 (3.67%)	14 (2.52%)	19 (3.47%)	
Educational attainment (%)						<0.001
Less than 9th Grade	146 (6.85%)	55 (10.24%)	39 (7.94%)	29 (5.22%)	23 (4.20%)	
9–11th Grade	209 (9.81%)	68 (12.66%)	37 (7.54%)	62 (11.15%)	42 (7.68%)	
High School Grad/GED or Equivalent	512 (24.03%)	151 (28.12%)	119 (24.24%)	130 (23.38%)	112 (20.48%)	
Some College or AA degree	657 (30.83%)	171 (31.84%)	164 (33.40%)	171 (30.76%)	151 (27.61%)	
College Graduate or above	607 (28.48%)	92 (17.13%)	132 (26.88%)	164 (29.50%)	219 (40.04%)	
Marital status (%)						<0.001
Married or living with partner	1,376 (64.57%)	310 (57.73%)	326 (66.40%)	353 (63.49%)	387 (70.75%)	
Divorced or living without partner	755 (35.43%)	227 (42.27%)	165 (33.60%)	203 (36.51%)	160 (29.25%)	
PIR (%)						<0.001
<1.3	436 (20.46%)	170 (31.66%)	107 (21.79%)	80 (14.39%)	79 (14.44%)	
1.3 ~ 3.5	832 (39.04%)	210 (39.11%)	207 (42.16%)	228 (41.01%)	187 (34.19%)	
>3.5	836 (40.50%)	157 (29.24%)	177 (36.05%)	248 (44.60%)	281 (51.37%)	
WtHR (%)						<0.001
≥0.5	1898 (89.07%)	500 (93.11%)	436 (88.80%)	504 (90.65%)	458 (83.73%)	
<0.5	233 (10.93%)	37 (6.89%)	55 (11.20%)	52 (9.35%)	89 (16.27%)	
Energy (kcal/day)	1914.43 ± 849.60	1372.11 ± 505.85	1711.67 ± 545.58	2073.69 ± 675.19	2572.15 ± 1025.47	<0.001
eGFR (mL/min/1.73 m^2^)	120.27 ± 26.95	119.47 ± 28.79	118.88 ± 28.63	119.56 ± 25.72	123.01 ± 24.53	0.013
Alcohol consumption (%)						0.103
Yes	328.00 (15.39%)	95.00 (17.69%)	84.00 (17.11%)	75.00 (13.49%)	74.00 (13.53%)	
No	1803.00 (84.61%)	442.00 (82.31%)	407.00 (82.89%)	481.00 (86.51%)	473.00 (86.47%)	
Smoking history (%)						0.008
Yes	1205.00 (56.55%)	349.00 (64.99%)	271.00 (55.19%)	305.00 (54.86%)	280.00 (51.19%)	
No	926.00 (43.45%)	188.00 (35.01%)	220.00 (44.81%)	251.00 (45.14%)	267.00 (48.81%)	
Heart Failure (%)						0.059
Yes	106 (4.97%)	38 (7.08%)	28 (5.70%)	24 (4.32%)	16 (2.93%)	
No	2025 (95.03%)	499 (92.92%)	463 (94.30%)	532 (95.68%)	531 (97.07%)	
Coronary heart disease (%)						0.019
Yes	200 (9.39%)	60 (11.17%)	59 (12.02%)	39 (7.01%)	42 (7.68%)	
No	1931 (90.61%)	477 (88.83%)	432 (87.98%)	517 (92.99%)	505 (92.32%)	
Stroke (%)						0.010
Yes	134 (6.29%)	51 (9.50%)	33 (6.72%)	30 (5.40%)	20 (3.66%)	
No	1997 (93.71%)	486 (90.50%)	458 (93.28%)	526 (94.60%)	527 (96.34%)	
Arthritis (%)						0.134
Yes	1,013 (47.54%)	277 (51.58%)	233 (47.45%)	253 (45.50%)	250 (45.70%)	
No	1,118 (52.46%)	260 (48.42%)	258 (52.55%)	303 (54.50%)	297 (54.30%)	
Hypertension (%)						0.004
Yes	1,111 (52.14%)	315 (58.66%)	260 (52.95%)	286 (51.44%)	250 (45.70%)	
No	1,020 (47.86%)	222 (41.34%)	231 (47.05%)	270 (48.56%)	297 (54.30%)	
Diabetes (%)						0.236
Yes	388 (18.21%)	108 (20.11%)	91 (18.53%)	103 (18.53%)	86 (15.72%)	
No	1743 (81.79%)	429 (79.89%)	400 (81.47%)	453 (81.47%)	461 (84.28%)	
All-cause mortality						<0.001
Yes	673 (31.58%)	194 (36.13%)	166 (33.81%)	175 (31.47%)	138 (25.23%)	
No	1,458 (68.42%)	343 (63.87%)	325 (66.19%)	381 (68.53%)	409 (74.77%)	
Cancer-cause mortality						<0.001
Yes	205 (12.33%)	74 (17.75%)	47 (12.63%)	50 (11.60%)	34 (7.67%)	
No	1,458 (87.67%)	343 (82.25%)	325 (87.37%)	381 (88.40%)	409 (92.33%)	
Cardiovascular mortality						0.472
Yes	146 (9.10%)	33 (8.78%)	37 (10.22%)	40 (9.50%)	36 (8.09%)	
No	1,458 (90.90%)	343 (91.22%)	325 (89.78%)	381 (90.50%)	409 (91.91%)	

Q1–Q4: Grouped by quartile according to OBS. Mean ± SD for continuous variables with normal distribution: *p*-value was calculated by *t*-test. Median [Interquartile range] for continuous variables with skewed distribution: *p*-value was calculated by Rao-Scott Chi-Square. % for categorical variables: *p*-value was calculated by Kruskal-Wallis test.

### The association between OBS and all-cause mortality, cancer-specific mortality, and CVD mortality among cancer survivors

**Figure 2:**
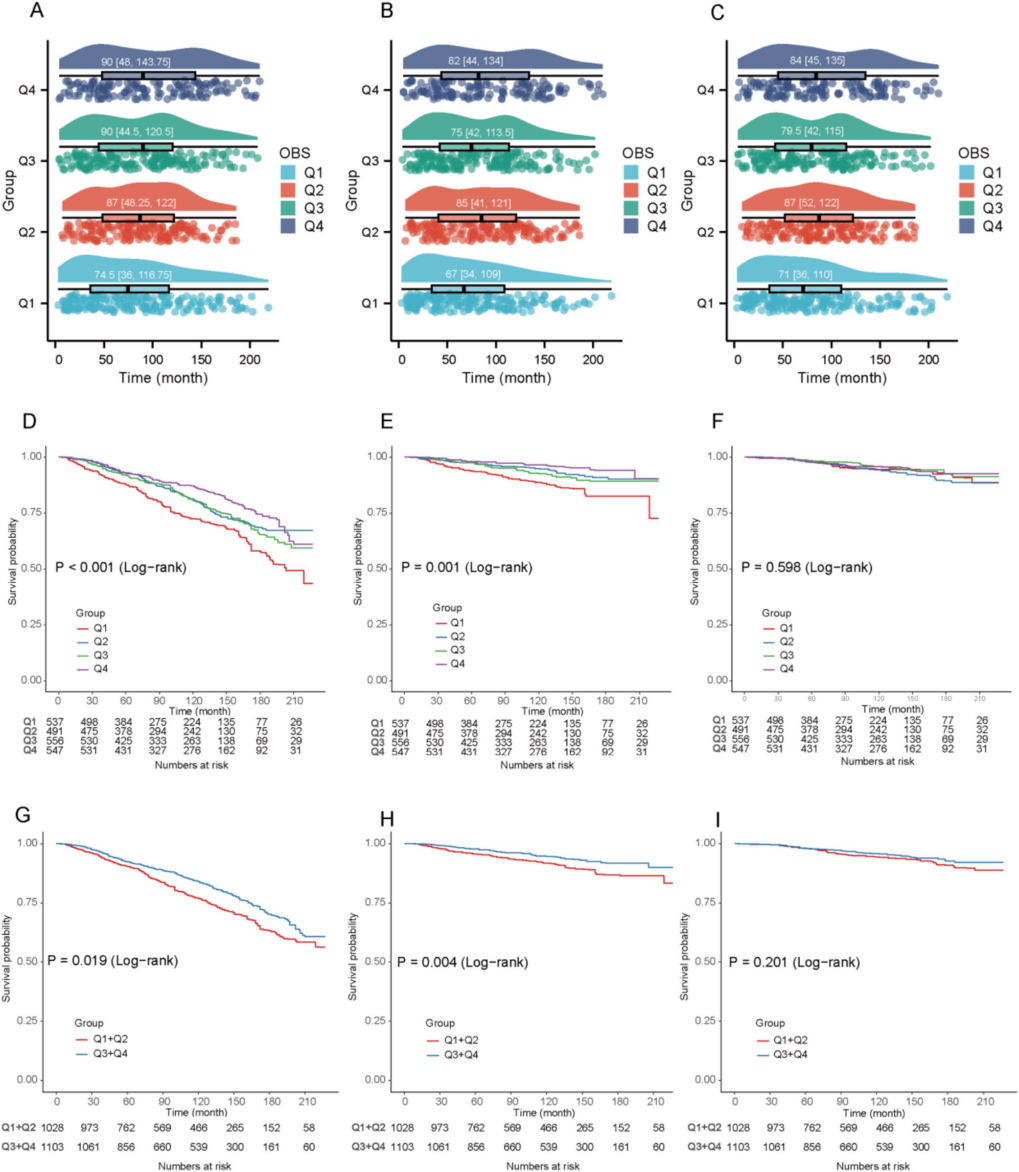
**(A)** The raincloud plot illustrates the distribution of survival times among participants who experienced all-cause mortality across different quartiles of OBS. **(B)** The raincloud plot illustrates the distribution of survival times among participants who experienced cancer-cause mortality across different quartiles of OBS. **(C)** The raincloud plot illustrates the distribution of survival times among participants who experienced CVD mortality across different quartiles of OBS. **(D)** KM Curves for all-cause mortality in cancer survivors with OBS quartiles. **(E)** KM Curves for cancer-cause mortality in cancer survivors with OBS quartiles. **(F)** KM Curves for CVD mortality in cancer survivors with OBS quartiles. **(G)** KM Curves for all-cause mortality in cancer survivors with OBS dichotomized groups. **(H)** KM Curves for cancer-cause mortality in cancer survivors with OBS dichotomized groups. **(I)** KM Curves for CVD mortality in cancer survivors with OBS dichotomized groups.

Raincloud plots illustrated the distribution of survival times across different quartiles of the OBS for individuals who died from all-cause (Figure 2A), cancer-cause (Figure 2B), and CVD mortality (Figure 2C), with median values and interquartile ranges clearly marked. Notably, participants in the lowest quartile (Q1) had the shortest median survival times across all three mortality categories. Furthermore, the median survival times for all-cause and cancer-cause deaths in Q1 and Q2 were generally shorter than those observed in Q3 and Q4. The KM survival curves indicate that cancer survivors in the highest OBS quartile have a significantly higher survival probability for both all-cause mortality and cancer-specific mortality compared to those in the lowest quartile (Log-rank *p* < 0.001, Figure 2D; Log-rank *p* = 0.001, Figure 2E). No significant difference was observed for CVD mortality (Log-rank *p* = 0.598, Figure 2F). To enhance the clarity of the KM curves and provide a more intuitive understanding of the association between OBS and survival, participants were grouped into low (Q1 + Q2) and high (Q3 + Q4) OBS categories. The analysis revealed that cancer survivors in the high OBS group exhibited significantly better survival outcomes for both all-cause mortality and cancer-cause mortality compared with those in the low OBS group (Log-rank *p* = 0.019, Figure 2G; Log-rank *p* = 0.004, Figure 2H). No significant difference was observed for CVD mortality between the two groups (Log-rank *p* = 0.201, Figure 2I).

**Figure 3:**
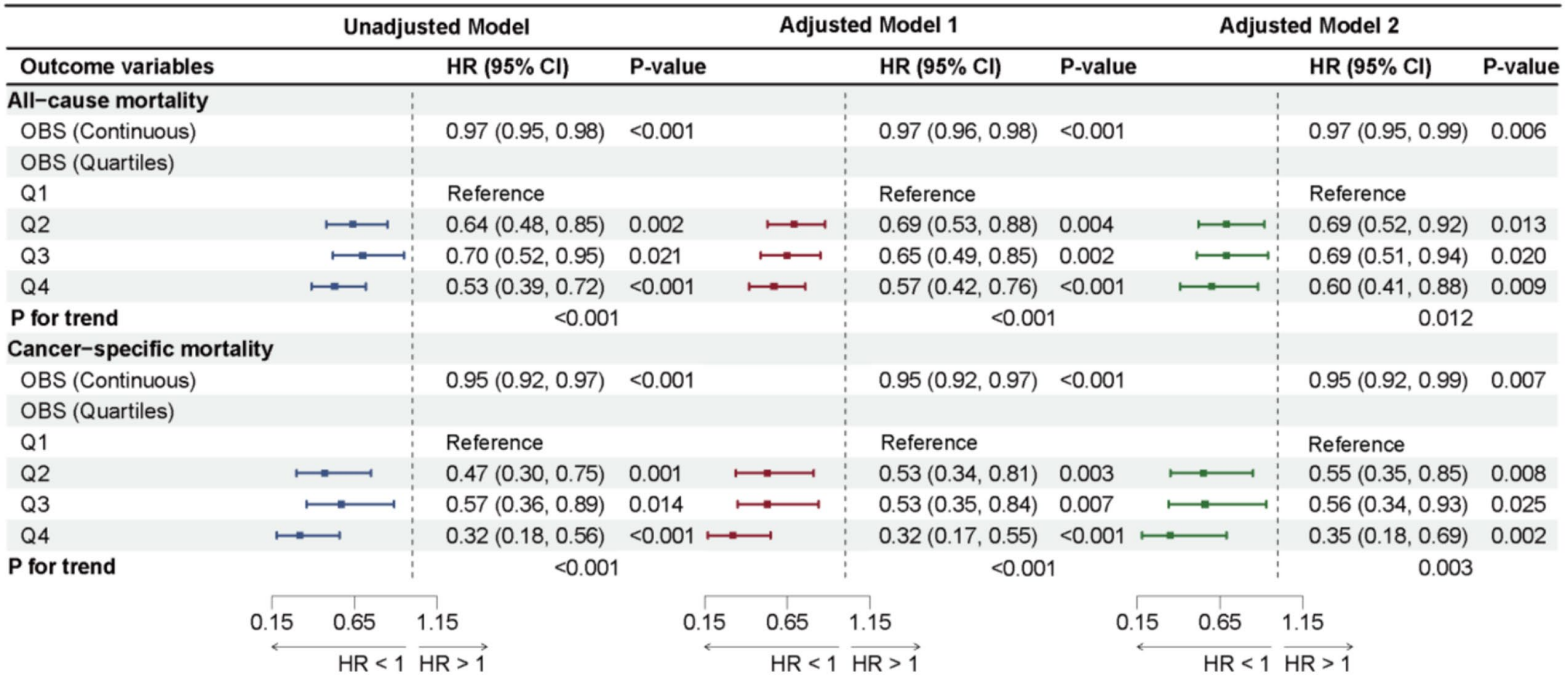
Forest plot illustrates the results of cox proportional hazards regression analyses for the association of OBS with all-cause mortality and cancer-cause mortality.

The results from the weighted Cox regression analysis show that in Adjusted Model 2, a one-unit increase in OBS among cancer survivors is associated with a 3% reduction in all-cause mortality (HR: 0.97, 95% CI: 0.95, 0.99, *p* = 0.006) and a 5% reduction in cancer-specific mortality (HR: 0.95, 95% CI: 0.92, 0.99, *p* = 0.007). The risk of all-cause mortality for the highest OBS group (Q4) is reduced by 40% compared to the lowest group (Q1) (HR: 0.60, 95% CI: 0.41, 0.88, *p* = 0.009), and the risk of cancer-specific death is reduced by 65% (HR: 0.35, 95% CI: 0.18, 0.69, *p* = 0.002), with significant trends for both all-cause and cancer-specific mortality (*p* for trend = 0.007; *p* for trend = 0.003). Significant statistical significance was also observed in Adjusted Model 1 and Unadjusted Model, as shown in Figure 3. However, while significant associations between OBS and CVD mortality were found no significant association was found in Unadjusted Model, Adjusted Model 1, and Adjusted Model 2 (*p* > 0.05). Similarly, no significant trends were observed across the three models, as shown in Supplementary Table 2.

Further analysis of larger cancer types in Adjusted Model 2 revealed a significant association between OBS and all-cause mortality risk among breast cancer survivors, with a one-unit increase in OBS associated to a 5% reduction in all-cause mortality (HR: 0.95, 95% CI: 0.91, 1.00, *p* = 0.038), while no statistically significant associations were found in subgroups of melanoma, non-melanoma skin cancer, prostate cancer, uterine or cervical cancers, and colorectal cancer in Adjusted Model 2 (all *p*-values > 0.05), detailed in Supplementary Table 3.

**Figure 4:**
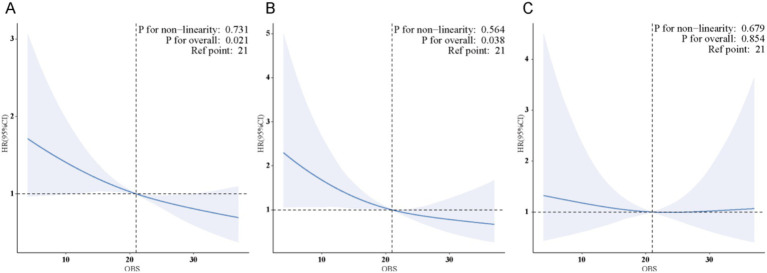
**(A)** There is a linear association between OBS and all-cause mortality in cancer survivors. **(B)** There is a linear association between OBS and cancer-specific mortality in cancer survivors. **(C)** No significant association was found between OBS and CVD mortality in cancer survivors.

The weighted RCS analysis indicated a linear negative association between OBS and both all-cause and cancer-specific mortality among cancer survivors, as shown in Figure 4.

### Subgroup analyses

**Figure 5:**
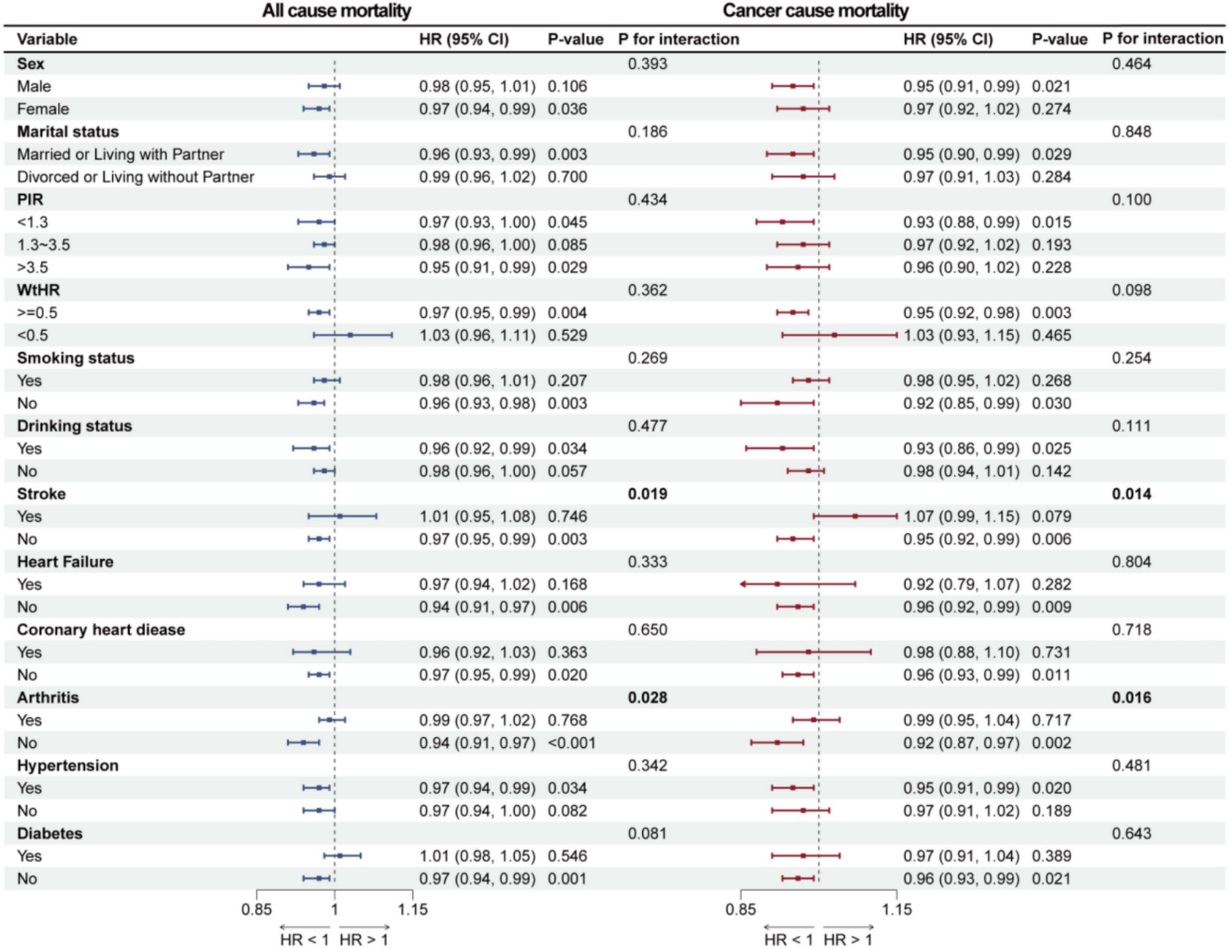
Forest plot illustrates the results of subgroup analyses for the association of OBS with all-cause mortality and cancer-cause mortality.

Subgroup analyses were conducted to explore whether the association between OBS and overall survival as well as cancer-specific survival among cancer survivors differs across various populations. Sex, marital status, PIR, WtHR, history of drinking, smoking, stroke, heart failure, coronary heart disease, arthritis, hypertension, and diabetes were considered as stratification factors for the weighted interaction tests. In the stratified analyses, the association between OBS and all-cause and cancer-cause mortality was statistically significant only among participants without a history of stroke or arthritis (*p* < 0.05), whereas this association was not significant among those with stroke or arthritis history (*p* > 0.05), and interaction analyses demonstrated significant heterogeneity between groups (*p* for interaction < 0.05), indicating that the effect of OBS on mortality risk significantly differed by the presence or absence of these conditions. Detailed results can be found in Figure 5.

### Sensitivity analyses

**Figure 6:**
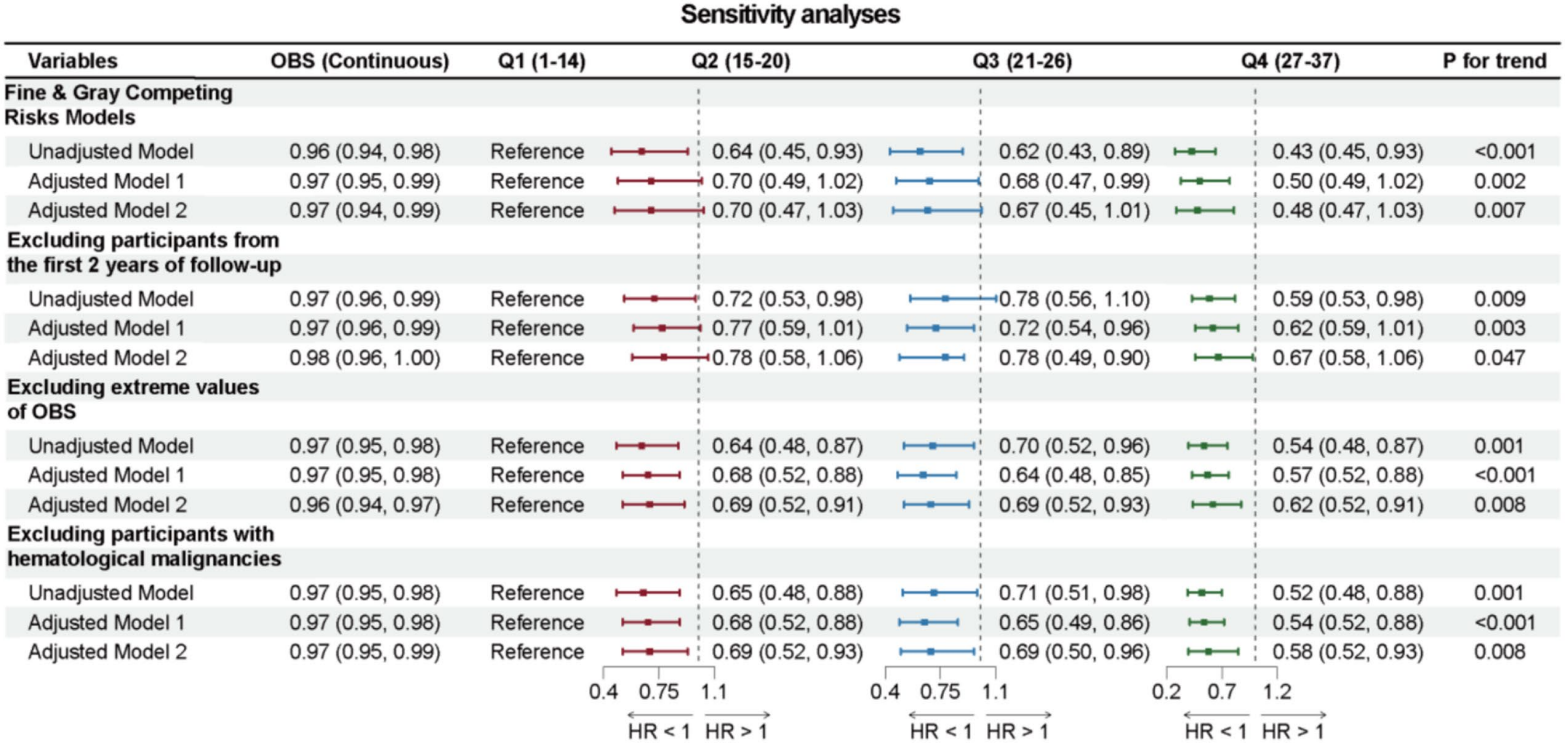
Forest plot illustrates the results of sensitivity analyses.

Sensitivity analyses were conducted to further validate the robustness of the main conclusions of this study. First, using Fine & Gray Competing Risks Models, the association between OBS and cancer-specific mortality also remained robust. Next, after excluding 76 cancer survivors who died within 24 months of follow-up, the association between OBS and reduced all-cause mortality remained significant across all three models. Moreover, even after excluding cancer survivors with hematologic malignancies, the association between OBS and all-cause mortality continued to be significant. Finally, after excluding 106 cancer survivors with extreme OBS (top and bottom 2.5%), the association between OBS and all-cause mortality continued to be significant, detailed in Figure 6.

## Discussion

This study presents a large-scale retrospective cohort analysis of 2,131 cancer survivors from the NHANES database covering the years 2001 to 2018. The findings reveal that higher OBS significantly associated with lower risks of all-cause and cancer-specific mortality among these survivors, demonstrating a clear linear negative association, although no significant association was found with CVD mortality. Notably, this association is especially strong among breast cancer survivors. Sensitivity analyses further support the reliability of these results.

Previous studies have indicated that consuming dietary antioxidants can lower all-cause mortality among cancer survivors (24, 25), while other research has shown that lifestyle changes—such as engaging in moderate exercise, managing weight, and quitting smoking—can also enhance inflammation and oxidative stress levels (26, 27). Researchers have reported a negative association between OBS and the risk of all-cause and cardiovascular mortality among individuals with diabetes or prediabetes (21). However, there is a notable gap in research that addresses cancer survivors, particularly studies that combine antioxidant diets with lifestyle factors. This study is pioneering in identifying a significant association between the OBS and both all-cause and cancer-specific mortality in cancer survivors. This finding suggests that variations in diet and lifestyle affecting oxidative balance have crucial implications for the prognosis of these individuals. Moreover, this study expands the applicability of OBS and suggests its potential utility in risk stratification for the long-term survival of cancer survivors.

The OBS, an emerging comprehensive measure of oxidative balance, has begun to demonstrate its clinical value in specific areas of cancer and other diseases. Longitudinal cohort studies indicate that the OBS may offer protective benefits for the long-term survival of breast and colorectal cancer patients (28). Moreover, significant associations have been established between the OBS and conditions such as non-alcoholic fatty liver disease, coronary heart disease, and metabolic syndrome (21, 22, 29). These chronic diseases are frequently observed among cancer survivors and can contribute to CVD mortality. Thus, there is strong theoretical backing for employing the OBS to evaluate all-cause and cause-specific mortality in cancer survivors.

Oxidative stress has gained significant attention in oncology. An imbalance between pro-oxidants and antioxidants can elevate the production of ROS through oxidative stress pathways. When ROS accumulates beyond a certain level, it can induce mitochondrial dysfunction and metabolic reprogramming, which are associated to mutations in oncogenes, loss of mitochondrial DNA, and activation of inflammatory pathways (30). The resulting disruptions in the cellular environment and lipid peroxidation are critical drivers of disease progression (31), ultimately contributing to cancer development (32, 33). Research indicates that exogenous antioxidants, such as vitamin C and *β*-carotene, play a role in maintaining cellular antioxidant defenses by scavenging ROS and reactive nitrogen species (RNS), likely by activating DNA repair mechanisms and inhibiting inflammatory pathways (34, 35). Additionally, studies show that smokers and those who consume alcohol tend to have significantly lower antioxidant levels and higher oxidative stress (36). In obese individuals, increased oxidative stress has been associated with RNS accumulation and ongoing chronic inflammation, resulting in mutations in both oncogenes and tumor suppressor genes (37). The regulation of oxidative balance in cancer survivors involves complex mechanisms and various influencing factors. However, most prior research has focused on these aspects individually. This study’s use of the OBS to evaluate all-cause and cause-specific mortality among cancer survivors allows for more comprehensive and accurate conclusions.

Oxidative stress has been well established as a critical driver in the progression of cardiovascular disease. Disruption of the redox balance between ROS production and clearance leads to the accumulation of reactive species such as superoxide and peroxynitrite. These molecules further amplify ROS generation via pathways like the NOX-2/mitochondrial axis, ultimately impairing endothelial function and promoting cardiovascular events (38). Moreover, excess ROS intensifies inflammation and damages cellular structures and functions. In response to these pathological conditions, the heart undergoes structural and functional remodeling, manifesting as myocardial ischemia–reperfusion injury, arrhythmias, and diabetic cardiomyopathy (39–41). In our study, we observed no significant link between OBS and cardiovascular mortality in cancer survivors. This may be explained by the fact that cardiovascular deaths in this population are predominantly driven by pre-existing health conditions. We offer the following analysis: for cancer survivors, CVD deaths are often associated to existing comorbidities. Moreover, oxidative imbalance leading to cancer recurrence or progression generally poses a greater mortality risk than chronic cardiovascular conditions such as hypertension or coronary heart disease. Additionally, researchers reviewing existing evidence on plant-based food intake, antioxidant supplementation, and their relationship with cardiovascular disease and cancer have found that taking one or more antioxidant supplements offers no clear preventive benefit for cardiovascular disease. Studies relying on dietary antioxidant intake tend to show weaker and more inconsistent associations with cardiovascular disease and cancer than those using biomarker-based assessments. Given that OBS is a composite score reflecting diet and lifestyle factors, this may have diluted its association with cardiovascular mortality (42). Notably, several factors may have contributed to the null findings. First, the follow-up period may have been insufficient to capture the long-term cumulative effects of OBS on cardiovascular outcomes, particularly given the slow and progressive nature of CVD. Second, potential misclassification of cause of death could have attenuated the observed associations. Although mortality data were derived from the National Death Index—a widely accepted and authoritative source—non-differential misclassification between cancer-related and cardiovascular deaths may still have occurred. Additionally, our subgroup analysis by cancer type revealed a statistically significant association for breast cancer. Past case–control studies have connected OBS, dietary OBS, and breast cancer risk (43–45). Research has shown that excessive ROS accumulation not only damages DNA and disrupts the tumor microenvironment—leading to hypoxia, inflammation, and immune suppression—but also contributes to breast cancer development by modulating hormone activity and promoting resistance to therapy through various pathways. Antioxidant strategies targeting estrogen oxidation have been found to reduce oxidative DNA damage and delay breast tumor onset (46). Specific antioxidants like tocopherol and zinc can lower IL-10 expression in breast tissue, selenium supports sustained glutathione peroxidase activity, while vitamin A and retinol can activate COX-2 gene transcription in breast stromal cells, collectively offering protective benefits to breast tissue (47). This study not only provides supportive evidence for previous findings but also suggests a significant association between OBS and all-cause mortality risk in breast cancer patients, which could underscore the importance of oxidative stress management in clinical care for breast cancer patients.

Subgroup analysis further revealed that the association between OBS and all-cause mortality as well as cancer-specific mortality was significant only among participants without stroke and arthritis, highlighting stroke and arthritis as key factors in differentiating mortality risks. In cancer survivors, stroke may involve imbalances in brain iron homeostasis and oxidative stress, both of which are crucial to tumor growth (48, 49). Stroke aftereffects often result in decreased nutritional status and quality of life, strongly associated to OBS. Research has also identified a association between arthritis and increased cancer risk, possibly due to shared risk factors and chronic inflammation (50). Furthermore, some antirheumatic treatments may disrupt the tumor immune microenvironment, impacting long-term survival in cancer survivors (51). Both stroke and arthritis are conditions involving persistent oxidative stress and widespread inflammation, and these shared pathological features may stem from disrupted oxidative balance (52).

In the sensitivity analyses, the association between higher OBS and reduced all-cause and cancer-specific mortality remained statistically significant after excluding outliers, participants with hematological malignancies, and individuals who died within the first two years of follow-up. Furthermore, when applying the Fine-Gray competing risk model to account for the competing risk of non-cancer death, the observed associations were consistent. These findings suggest the robustness of the association between oxidative balance and mortality outcomes. A possible explanation is that the biological mechanisms underlying the protective role of antioxidant-rich status are relatively stable and less likely to be influenced by outliers or short-term fatal conditions.

### Strengths and limitations

The strengths of this study include its large-sample retrospective cohort design, weighted analysis, and adjustment for multiple confounders. This approach allowed for the first identification of a significant linear negative association between OBS and both all-cause and cancer-specific mortality risks in cancer survivors, with no significant association found with CVD mortality. Additionally, breast cancer patients showed stronger statistical significance within cancer subtype analyses. Stroke and arthritis were identified as key stratifying factors for all-cause and cancer-specific mortality. Sensitivity analyses further confirmed the robustness of these findings.

Despite its strengths, this study has several limitations. First, while it included a sufficient number of covariates, the observational nature of the study may still allow for potential confounding factors. Second, the NHANES database does not capture information on the pathological types, clinical stages, or specific treatment modalities of different tumors. This limitation not only restricts the feasibility of more granular subgroup analyses but may also introduce residual confounding into the study findings. Furthermore, although multiple imputation was employed to address missing data, this method relies on the assumption that data are missing at random, any violation of this assumption could introduce bias of our findings. Imputed values are inherently estimated rather than observed, which may introduce uncertainty into the analysis and limit the precision of our effect estimates. Additionally, data loss from long-term follow-up may introduce selection bias and the OBS reflects the status of cancer survivors at a single point in time, and there is insufficient follow-up and repeated assessment data to confirm the robustness of the findings. Finally, the inherent limitations of an observational study design preclude definitive causal interpretations of the association between OBS and mortality risk.

## Conclusion

The study revealed that among cancer survivors, higher OBS are significantly associated to lower risks of both all-cause and cancer-specific mortality, while no such association was noted for CVD mortality. This inverse association is especially evident in survivors who do not have a history of stroke or arthritis. These findings suggest that cancer survivors might improve their chances of survival by managing elevated OBS levels to help sustain bodily oxidative balance.

## Data Availability

Publicly available datasets were analyzed in this study. This data can be found at: All data used in this study were sourced from NHANES, www.cdc.gov/nchs/NHANEs/.

## Footnotes

^1^ https://www.cdc.gov/nchs/nhanes/
