# Supplementary material for: The association between oxidative balance scores and all-cause mortality and cancer-specific mortality in cancer survivors: a retrospective cohort study
Source: Front Nutr. 2025 May 21;12:1522048. doi: 10.3389/fnut.2025.1522048 (PMC12133489; doi:10.3389/fnut.2025.1522048)
Supplement: Supplementary file 1 [file Table_1.doc]

Supplementary Table 1 The components of OBS[1][2]

| ­­­­­ | Male | | | Female | | | Property |
| --- | --- | --- | --- | --- | --- | --- | --- |
|  | 0 | 1 | 2 | 0 | 1 | 2 |  |
| **Dietary OBS components** |  |  |  |  |  |  |  |
| Dietary fiber (g/d) | <12.70 | 12.80-20.50 | >20.60 | <10.05 | 10.60-16.80 | >16.90 | A |
| Carotene (RE/d) | <457.00 | 460.00-777.00 | >778.00 | <346.00 | 347.00-655.00 | >660.00 | A |
| Riboflavin (mg/d) | <1.83 | 1.83-2.62 | >2.64 | <1.40 | 1.40-2.09 | >2.09 | A |
| Niacin (mg/d) | <19.17 | 19.18-28.26 | >28.32 | <14.53 | 14.55-21.72 | >21.73 | A |
| Vitamin B6 (mg/d) | <1.57 | 1.57-2.39 | >2.41 | <1.16 | 1.16-1.82 | >1.82 | A |
| Total folate (mcg/d) | <303.00 | 306.00-472.00 | >473.00 | <248.00 | 248.00-382.00 | >383.00 | A |
| Vitamin B12 (mcg/d) | <3.20 | 3.22-6.12 | >6.14 | <2.40 | 2.41-4.64 | >4.65 | A |
| Vitamin C (mg/d) | <40.00 | 40.20-107.20 | >108.40 | <31.50 | 31.60-94.30 | >94.40 | A |
| Vitamin E (ATE) (mg/d) | <5.35 | 5.36-8.87 | >8.91 | <4.27 | 4.28-7.38 | >7.39 | A |
| Calcium (mg/d) | <635.00 | 637.00-1053.00 | >1063.00 | <558.00 | 559.00-895.00 | >898.00 | A |
| Magnesium (mg/d) | <242.00 | 243.00-347.00 | >348.00 | <193.00 | 194.00-289.00 | >290.00 | A |
| Zinc (mg/d) | <8.82 | 8.90-13.60 | >13.67 | <6.64 | 6.65-10.32 | >10.34 | A |
| Copper (mg/d) | <1.01 | 1.01-1.47 | >1.47 | <0.83 | 0.83-1.22 | >1.22 | A |
| Selenium (mcg/d) | <85.70 | 86.10-128.00 | >128.10 | <65.90 | 66.10-98.40 | >98.50 | A |
| Total fat (g/d) | >95.96 | 61.56-95.95 | <61.45 | >72.07 | 47.80-71.86 | <47.77 | P |
| Iron (mg/d) | >18.32 | 12.15-18.24 | <12.14 | >14.34 | 9.40-14.30 | <9.39 | P |
| **Lifestyle OBS components** |  |  |  |  |  |  |  |
| Physical activity (MET-minute/week) | <840.00 | 858.67-2709.00 | >2730.00 | <720.00 | 724.50-2060.33 | >2072.00 | A |
| Alcohol (drinks/d) | >30 | 0-30 | None | >15 | 0-15 | None | P |
| Body mass index (kg/m2) | >29.62 | 25.81-29.60 | <25.80 | >30.62 | 24.98-30.60 | <24.97 | P |
| Cotinine (ng/mL) | >0.09 | 0.02-0.08 | <0.02 | >0.10 | 0.02-0.09 | <0.02 | P |
| A: antioxidant; P: prooxidant; RE: retinol equivalent; ATE: alpha-tocopherol equivalent; MET: metabolic equivalent. | | | | | | | |

**References:**

1. Hernández-Ruiz Á, García-Villanova B, Guerra-Hernández EJ, et al. Oxidative Balance Scores (OBSs) Integrating Nutrient, Food and Lifestyle Dimensions: Development of the NutrientL-OBS and FoodL-OBS. Antioxidants (Basel). 2022;11(2):300.
2. Zhang W, Peng SF, Chen L, Chen HM, Cheng XE, Tang YH. Association between the Oxidative Balance Score and Telomere Length from the National Health and Nutrition Examination Survey 1999-2002. Oxid Med Cell Longev. 2022;2022:1345071.

**Supplementary Table 2** Cox proportional hazards regression analyses for the association of OBS with CVD mortality

| Outcome variables | Unadjusted Model | Adjusted Model 1 | Adjusted Model 2 |
| --- | --- | --- | --- |
|  | HR (95%CI), P-value | | |
| OBS | 0.97 ( 0.95, 1.00), 0.067 | 0.97 ( 0.94, 1.00), 0.089 | 1.00 ( 0.96, 1.04), 0.983 |
| OBS (Quartiles) |  |  |  |
| Q1 | Reference | Reference | Reference |
| Q2 | 1.16 (0.67, 2.00), 0.601 | 1.15 (0.68, 1.94), 0.593 | 1.47 (0.83, 2.62), 0.190 |
| Q3 | 0.82 (0.47, 1.45), 0.503 | 0.71 (0.41, 1.23), 0.221 | 1.13 (0.58, 2.20), 0.713 |
| Q4 | 0.83 (0.44, 1.55), 0.560 | 0.86 (0.43, 1.72), 0.668 | 1.62 (0.69, 3.84), 0.269 |
| Unadjusted Model: no covariate adjustments. | | | |
| Adjusted Model 1: adjusted for age, sex, race, marital status, education, and poverty to income ratio. | | | |
| Adjusted Model 2: adjusted for age, sex, race, marital status, education poverty to income ratio, eGFR, energy intake, waist-to-height ratio, alcohol consumption, smoking history, hypertension, diabetes, stroke, coronary heart disease, heart failure, and arthritis. | | | |

**Supplementary Table 3** Cox proportional hazards regression analyses for the association of OBS with all-cause mortality in cancer subgroups

| Outcome variables | Unadjusted Model | Adjusted Model 1 | Adjusted Model 2 |
| --- | --- | --- | --- |
|  | HR (95%CI), *P*-value | | |
| Cancer subgroups |  |  |  |
| Melanoma | 1.00 ( 0.94, 1.07), 0.926 | 1.04 ( 0.98, 1.11), 0.213 | 1.02 ( 0.90, 1.15), 0.751 |
| Non-melanoma skin cancer | 0.95 ( 0.93, 0.98), <0.001 | 0.96 ( 0.93, 0.98), 0.003 | 0.97 ( 0.93, 1.01), 0.128 |
| Prostate cancer | 0.98 ( 0.95, 1.00), 0.092 | 0.99 ( 0.96, 1.02), 0.459 | 1.00 ( 0.95, 1.04), 0.826 |
| Breast cancer | 0.96 ( 0.93, 1.00), 0.046 | 0.97 ( 0.94, 1.00), 0.055 | 0.95 ( 0.91, 1.00), 0.038 |
| Uterine and cervical cancers | 0.96 ( 0.89, 1.03), 0.254 | 0.94 ( 0.88, 1.00), 0.055 | 0.95 ( 0.88, 1.03), 0.247 |
| Colorectal cancer | 0.97 ( 0.93, 1.01), 0.188 | 0.96 ( 0.90, 1.03), 0.258 | 0.95 ( 0.87, 1.03), 0.231 |
| Unadjusted Model: no covariate adjustments. | | | |
| Adjusted Model 1: adjusted for age, sex, race, marital status, education, and poverty to income ratio. | | | |
| Adjusted Model 2: adjusted for age, sex, race, marital status, education poverty to income ratio, eGFR, energy intake, waist-to-height ratio, alcohol consumption, smoking history, hypertension, diabetes, stroke, coronary heart disease, heart failure, and arthritis. | | | |
